# Expression of HO1 and PER2 can predict the incidence of delirium in trauma patients with concomitant brain injury

**DOI:** 10.1038/s41598-021-94773-6

**Published:** 2021-07-28

**Authors:** Matti Steimer, Sandra Kaiser, Felix Ulbrich, Johannes Kalbhenn, Hartmut Bürkle, Nils Schallner

**Affiliations:** 1 Department of Anesthesiology & Critical Care Medicine, Medical Center - Faculty of Medicine, University of Freiburg, Hugstetter Str. 55, 79106 Freiburg, Germany; 2 Faculty of Medicine, University of Freiburg, Freiburg, Germany

**Keywords:** Biomarkers, Circadian rhythms and sleep, Translational research, Neurological manifestations

## Abstract

Intensive care unit (ICU)-acquired delirium is associated with adverse outcome in trauma patients with concomitant traumatic brain injury (TBI), but diagnosis remains challenging. Quantifying circadian disruption by analyzing expression of the circadian gene *period circadian regulator 2* (*PER2*) and *heme oxygenase 1* (*HO1*), which determines heme turnover, may prove to be potential diagnostic tools. Expression of *PER2* and *HO1* was quantified using qPCR from blood samples 1 day and 7 days after trauma. Association analysis was performed comparing mRNA expression levels with parameters of trauma (ISS—injury severity score), delirium, acute kidney injury (AKI) and length of ICU stay. 48 polytraumatized patients were included (equal distribution of TBI versus non-TBI) corrected for ISS, age and gender using a matched pairs approach. Expression levels of *PER2* and *HO1* were independent of age (*PER2*: *P* = 0.935; *HO1*: *P* = 0.988), while expression levels were significantly correlated with trauma severity (*PER2*: *P* = 0.009; *HO1*: *P* < 0.001) and longer ICU length of stay (*PER2*: *P* = 0.018; *HO1*: *P* < 0.001). High expression levels increased the odds of delirium occurrence (*PER2*: *OR* = 4.32 [1.14–13.87]; *HO1*: *OR* = 4.50 [1.23–14.42]). Patients with TBI showed a trend towards elevated *PER2* (*OR* = 3.00 [0.84–9.33], *P* = 0.125)*,* but not towards delirium occurrence (*P* = 0.556). TBI patients were less likely to develop AKI compared to non-TBI (*P* = 0.022). Expression levels of *PER2* and *HO1* correlate with the incidence of delirium in an age-independent manner and may potentially improve diagnostic algorithms when used as delirium biomarkers.

**Trial registration:** German Clinical Trials Register (Trial-ID DRKS00008981; Universal Trial Number U1111-1172-6077; Jan. 18, 2018).

## Introduction

Trauma patients in the ICU often exhibit impaired sleep–wake-cycles^1^, impaired cognition and impaired organ function, which might become manifest clinically as delirium and acute kidney injury (AKI). Disruption in circadian rhythm is now believed to play a major role in development of delirium^2^ and contribute to the pathogenesis of kidney disease^3,4^. Both delirium and AKI are independently associated with adverse patient outcome and mortality^5,6^. Their diagnosis however remains challenging^7,8^. A better physiological and biochemical understanding of delirium and AKI development is key to finding objective laboratory parameters to predict, diagnose and prognosticate outcome. Particularly in the study of delirium, multiple potential biomarkers in both serum and cerebrospinal fluid have been proposed^9–12^, however none have been shown to provide added clinical value^11^. Despite the proposed role of disrupted circadian rhythmicity in development of delirium the molecular machinery behind it has not been investigated in search of a biomarker yet.

The core clock genes that regulate autonomous circadian rhythm include the Period genes (Per 1–3)^13^. These are associated with susceptibility to organ dysfunction, including the brain and cardiovascular system^14–17^. Eckle et al. for instance identified transcriptional and post-translational regulation of *PER2* as well as the stabilization of the resulting protein as an integral step in the adaption of cardiomyocytes to ischemia. Consequently, *PER2*^*−/−*^ mice had larger infarct sizes than their wild-type controls. With regard to delirium, increasing *PER2* expression has been shown to be effective against midazolam-induced delirium in mice^18^.

The cytoprotective enzyme heme oxygenase 1 (HO1) serves as the rate-limiting step in heme metabolism and is strongly upregulated following various cellular stressors^19^. HO1 catalyzes the degradation of heme into iron, biliverdin and CO. Biliverdin is then further reduced to bilirubin by oxidation of NAD(P)H to NAH(P)^+^. *HO1* expression may serve as a potential predictive blood biomarker since it is closely linked to circadian regulation as it: (a) catabolizes the heme moiety contained in the circadian regulatory proteins NPAS-2 and RevErb-α, and (b) produces carbon monoxide (CO) as a bioactive product. Endogenous CO in turn modulates the transcriptional activity of NPAS-2, RevErb-α and CLOCK that control expression of *PER2*^20^. Moreover, antioxidant qualities of HO1 (and CO) are well documented. Especially in the case of AKI *HO1* induction has been identified as a rapid cytoprotective response in multiple animal models of AKI^21–26^.

Here, we hypothesized that *PER2* and *HO1* expression could serve as potential serum biomarkers to diagnose and predict delirium and/or AKI. We posited that alterations in *PER2* and *HO1* mRNA expression would indicate circadian disruption. Being at high risk to develop both delirium and AKI^27,28^, we tested this hypothesis in polytraumatized patients. As circadian rhythm is controlled by the hypothalamus, a trauma patient collective afforded us the opportunity to simultaneously investigate the effect of traumatic brain injury (TBI) on circadian disruption and clinical outcome. We studied a sub-group of patients with TBI and measured *PER2* and *HO1* mRNA expression and asked if expression correlated with subjects developing delirium and/or AKI.

## Results

### Patient characteristics and recruitment

**Figure 1:**
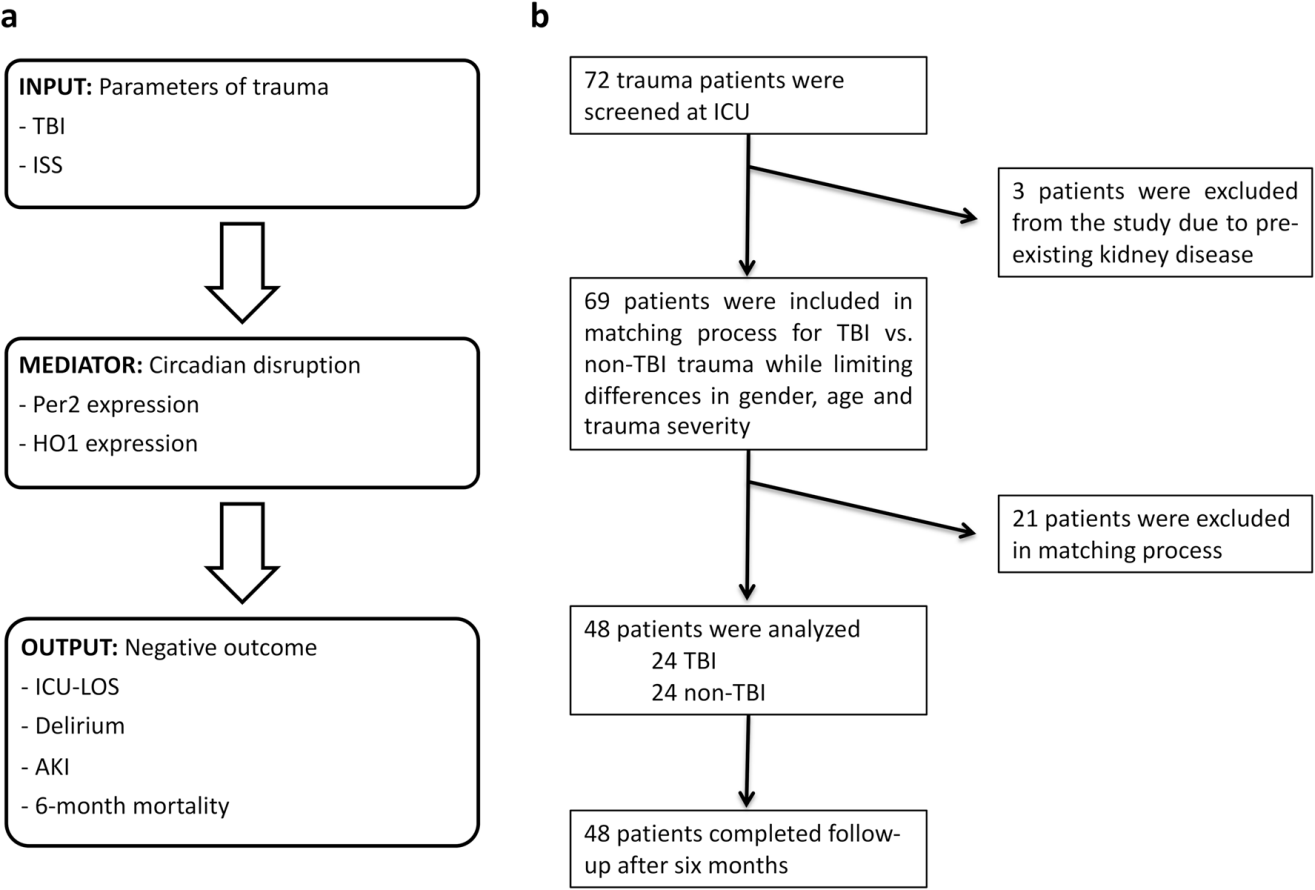
Study conception. **(a)** Schematic illustration of hypothesized relationship between input (parameters of trauma) and output (negative outcome) parameters with mediating variables (circadian disruption and heme metabolism measured via *PER2/HO1* expression). **(b)** Selection of polytrauma patients that were analyzed for circadian mRNA expression. Patients were sorted in matched pairs (criteria: gender, age, trauma severity (*ISS* injury severity score)) to form patient groups of TBI and non-TBI for later between-group comparison. *TBI* traumatic brain injury, *ISS* injury severity score, *PER2* period circadian regulator 2, *HO1* heme oxygenase 1, *AKI* acute kidney injury, *ICU* intensive care unit, *LOS* length of stay.

A total of 72 patients were screened of which three patients were excluded due to pre-existing kidney disease. Of the remaining 69, a total of 48 polytrauma patients were included in the study (Fig. 1b). Using a matched pairs approach, two 24-patient groups with TBI and non-TBI patients were formed. These groups did not significantly vary regarding age at trauma, gender, trauma ISS, pre-existing cognitive or cardiovascular disease, diabetes mellitus, time spent in the ICU and maximum leucocyte counts during the first week after trauma (Table 1).

**Table 1:** Patient characteristics.

n	Total	TBI	Non-TBI	*P*
	48	24	24	
**Age [years]**				
Mean	53.54	55.13	51.96	0.422
SD	16.73	17.70	15.92	
< 40	8	4	4	
40–75	36	18	18	
> 75	4	2	2	
**Gender**				
Male	42	21	21	
Female	6	3	3	
**Injury severity score**				
Mean	32.52	32.71	32.33	0.947
SD	12.60	13	12.46	
< 25	16	8	8	
25–36	20	10	10	
> 36	12	6	6	
Pre-existing neurocognitive disorder	4	3	1	0.609
Pre-existing cardiovascular disease	19	10	9	> 0.999
Diabetes mellitus	6	4	2	0.666
**ICU length of stay [days]**				
Mean	11.63	12.13	11.13	0.770
SD	11.25	11.82	10.62	
**Max. leucocyte count first week [1000/µl]**				
Median	14.54	13.78	15.20	0.617
IQR	[11.24 to 17.25]	[11.13 to 15.96]	[11.36 to 18.64]	

Presented in total and within matched groups. *P*-values of between-group comparisons of patients with traumatic brain injury (TBI) and without (non-TBI) calculated by Mann Whitney or Fisher’s exact test. All *P* > 0.05.

*SD* standard deviation, *IQR* inter-quartile ratio, *ICU* intensive care unit.

### *HO1* expression correlates with bilirubin concentration

**Figure 2:**
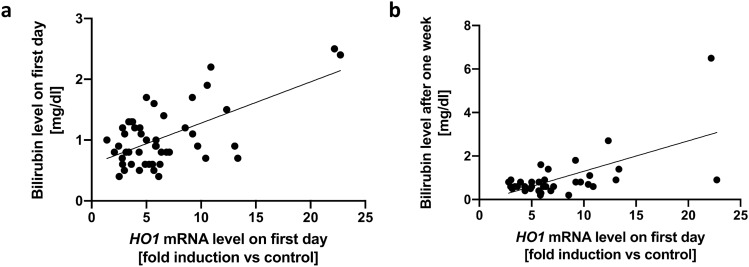
Correlation of systemic *HO1* expression with bilirubin plasma concentration. Linear regression curves of systemic *HO1* expression on admission and **(a)** bilirubin concentration on admission (Spearman: r = 0.315, *P* = 0.029, Linear Regression: r^2^ = 0.376, *P* < 0.0001) and (**b)** bilirubin expression after 1 week (Spearman: r = 0.424, *P* = 0.011, Linear Regression: r^2^ = 0.377, *P* < 0.0001). *HO1* heme oxygenase 1.

To verify that expression levels mirror actual enzymatic activity, we tested *HO1* expression and bilirubin concentration for correlation, as bilirubin is the final product of heme degradation. Indeed, *HO1* expression levels on the first day correlated with concurrent bilirubin concentration (r = 0.315, *P* = 0.029, r^2^ = 0.378, *P* < 0.001, Fig. 2a) on day one and after one week (r = 0.424, *P* = 0.011, r^2^ = 0.377, *P* < 0.001, Fig. 2b). The correlation of *HO1* expression after one week and bilirubin concentration at the same time missed significance (r = 0.253, *P* = 0.149, r^2^ = 0.100, *P* < 0.067, data not shown).

### Biomarkers correlate with trauma severity and duration of ICU treatment

**Figure 3:**
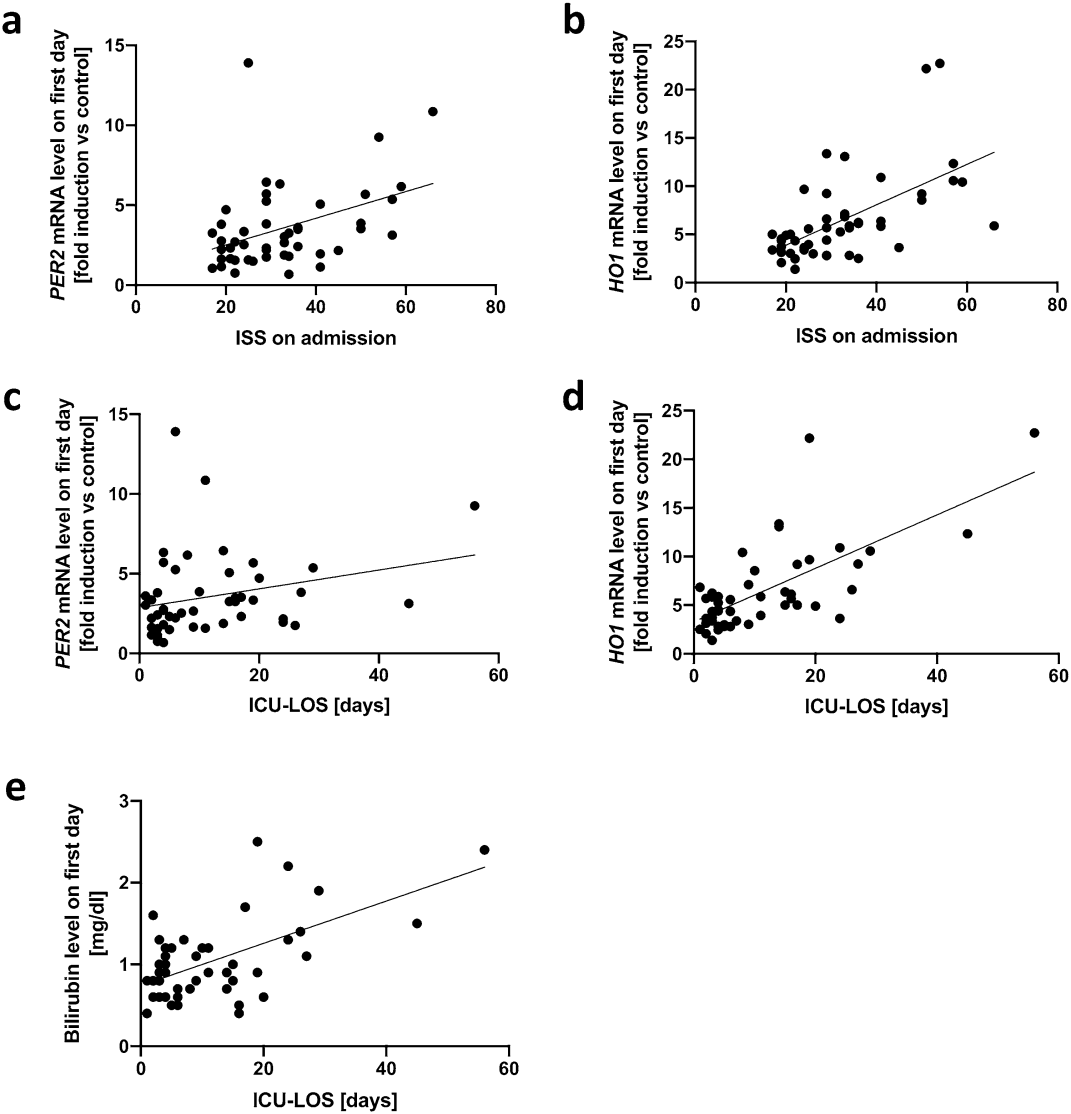
Correlation of systemic *PER2* and *HO1* expression with trauma severity and length of ICU stay. Linear regression curves of **(a)** patients’ ISS (trauma severity) and *PER2* expression (Spearman: r = 0.375, *P* = 0.009, Linear Regression: r^2^ = 0.166, *P* = 0.004), (**b)** patients’ ISS and *HO1* expression (r = 0.603, *P* < 0.001, r^2^ = 0.346, *P* < 0.001), **(c)** *PER2* expression and time spent in the ICU (r = 0.340, *P* = 0.018, r^2^ = 0.067, *P* = 0.008), **(d)** *HO1* expression and time spent in the ICU (r = 0.604, *P* < 0.001, r^2^ = 0.483, *P* < 0.001) and **(e)** bilirubin concentration and time spent in the ICU (r = 0.420, *P* = 0.003, r^2^ = 0.349, *P* < 0.001). *PER2* period circadian regulator 2, *HO1* heme oxygenase 1, *ISS* injury severity score, *ICU* intensive care unit.

To review our hypotheses (Fig. 1a), the relationships between input variable and mediator as well as between mediator and output data were tested. Indeed, a higher ISS correlated with significantly higher mRNA expression levels of *PER2* (r = 0.375, *P* = 0.009, r^2^ = 0.166, *P* = 0.004, Fig. 3a) and *HO1* (r = 0.603, P < 0.001, r^2^ = 0.346, P < 0.001, Fig. 3b), and among each other, (r = 0.424, *P* = 0.003, r^2^ = 0.144, *P* = 0.008, data not shown). Similarly, higher *PER2* (r = 0.340, *P* = 0.018, r^2^ = 0.067, *P* = 0.008, Fig. 3c) and *HO1* mRNA expression levels (r = 0.604, *P* < 0.001, r^2^ = 0.483, *P* < 0.0001, Fig. 3d) correlated with significantly longer ICU length of stay. Bilirubin concentration on day one showed no significant correlation with the ISS (r = 0.182; *P* = 0,216, data not shown). Higher bilirubin concentration on day one did, however, correlate significantly with longer ICU length of stay (r = 0.420; *P* = 0,003; r^2^ = 0.349, *P* < 0.001, Fig. 3e).

### Biomarkers correlate with occurrence of delirium

**Figure 4:**
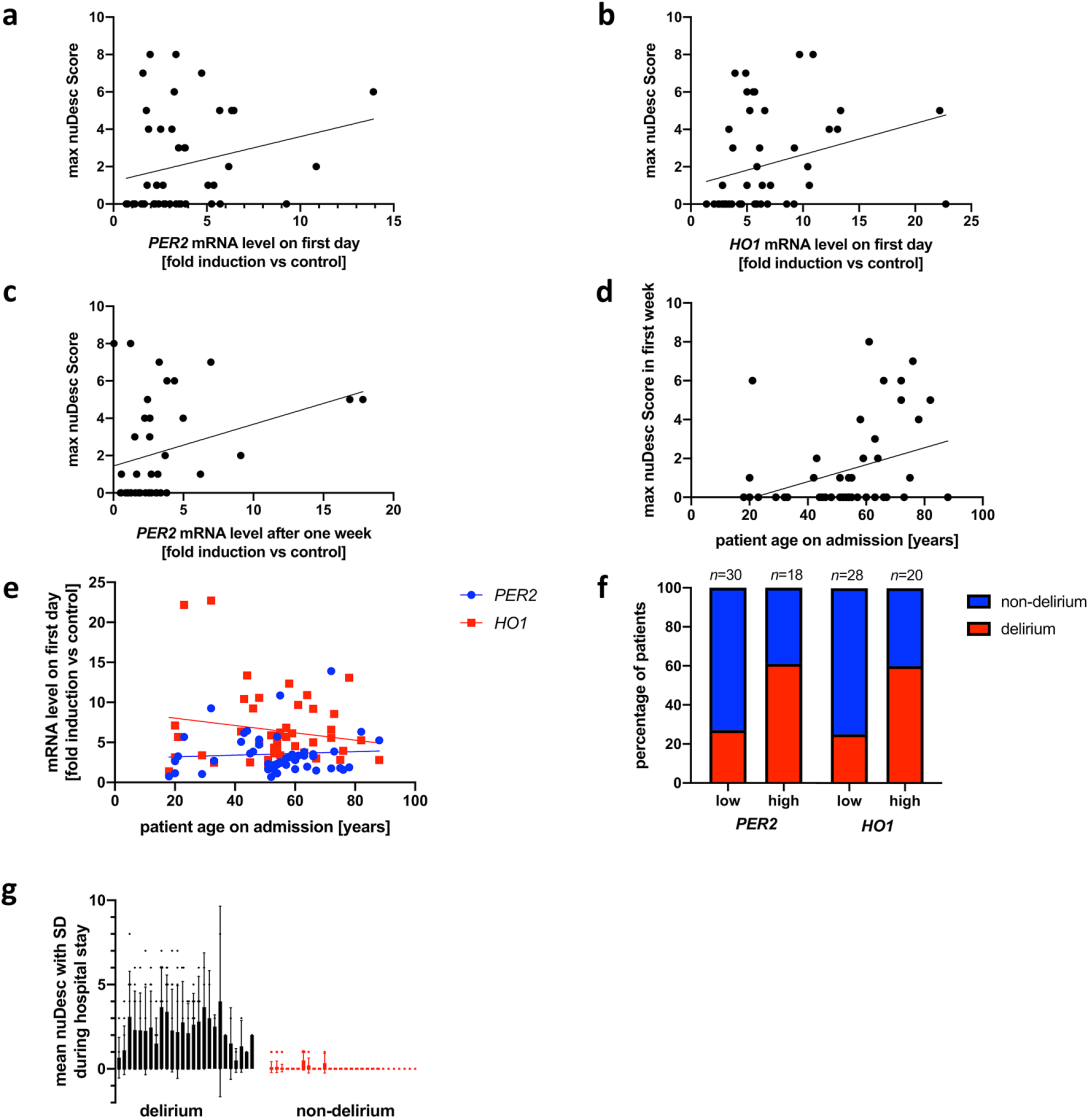
Correlation of *PER2* and *HO1* expression with delirium during hospitalization. **(a–e)** Linear regression curves between mRNA expression (*PER2/HO1*) as well as patients’ age at admission and maximal nuDesc. **(a,b)** Correlation of *PER2* (Spearman: r = 0.319, *P* = 0.027, Linear Regression: r^2^ = 0.057, *P* = 0.104) and *HO1* expression (r = 0.409, *P* = 0.004, r^2^ = 0.075, *P* = 0.080) on first day of trauma with their maximal nuDesc during hospitalization. **(c)** Correlation of *PER2* after 1 week and with their maximal nuDesc during hospitalization (r = 0.366, *P* = 0.024, r^2^ = 0.083, *P* = 0.048). **(d)** Correlation of age at admission with the maximal nuDesc during the first week of hospitalization (r = 0.370, *P* = 0,010, r^2^ = 0.105, *P* = 0.024). **(e) (**Absent) Correlation between patients’ age at admission and *PER2* (Spearman: *P* = 0.935, Linear Regression: *P* = 0.634) and *HO1* expression (*P* = 0.988, *P* = 0.247) on the first day of trauma. **(f)** Contingency diagrams analyzed via Fisher’s exact test showing distribution of delirium/non-delirium between patient groups of high and low mRNA expression levels of *PER2* (Fisher’s exact test: *P* = 0.032, Koopman asymptotic score: *RR* = 2.92, 95% CI 1.15–4.64; Baptista-Pike: *OR* = 4.32, 95% CI 1.14–13.87) and *HO1* (*P* = 0.019, *RR* = 2.40, 95% CI 1.18–5.07; *OR* = 4.50, 95% CI 1.23–14.42). Categories of high and low expression levels of *PER2* and *HO1* were formed from linear regression of ISS and mRNA expression levels on first day of trauma (cf. Fig. 3a,b). **(g)** Higher individual dispersion of nuDesc during hospital stay in patients of the category “delirium” than “non-delirium” (Mann–Whitney-U test: *P* < 0.0001, cf. Methods/clinical assessment). *nuDesc* nursing delirium screening scale, *95% CI* 95% confidence interval, *OR* odds ratio, *PER2* period circadian regulator 2, *HO1* heme oxygenase 1, *ISS* injury severity score.

A significant correlation was discovered between the expression of both *PER2* and *HO1* on day one after trauma and the maximum nuDesc (*PER2*: r = 0.319, *P* = 0.027, Fig. 4a; *HO1*: r = 0.409, *P* = 0.004, Fig. 4b). Correlation of delirium and *PER2* expression remained significant 1 week post trauma (r = 0.366, *P* = 0.024, r^2^ = 0.083, *P* = 0.048, Fig. 4c). In accordance with age as a major risk factor for the development of delirium^29^, maximum nuDesc in the first week after trauma correlated significantly with a patient’s age at the time of trauma (r = 0.370, *P* = 0.010, r^2^ = 0.105, *P* = 0.024, Fig. 4d). No correlation could be found between a patient’s age at the time of trauma and PER2 and HO-1 expression (*PER2*: r = 0.012, *P* = 0.935, r^2^ = 0.005, *P* = 0.634; *HO1*: r = 0.002, *P* = 0.988, r^2^ = 0.029, *P* = 0.247, Fig. 4e), suggesting an age-independent correlation between these gene expression patterns and delirium. Trauma severity measured by ISS did not correlate with maximum nuDesc after trauma (r = 0.070, *P* = 0.638, r^2^ = 0.001, *P* = 0.825, data not shown). Mean values of *PER2* and HO1 mRNA expression on day one after trauma (*P* = 0.013) and after one week (*P* = 0.016 and *P* = 0.004 respectively) were significantly elevated in delirious patients (Table 2). When dividing patients into categories of high and low mRNA expression in relation to ISS scoring, delirium (nuDesc > 1) was significantly more frequent in patients within the group of high *PER2* (*P* = 0.032, Fig. 4f) and *HO1* (*P* = 0.019, Fig. 4f) expression. Relative risk to develop delirium was 2.92 (95% CI 1.15–4.64) for patients with high *PER2* and 2.40 (95% CI 1.18–5.07) for patients high *HO1* expression. Correspondingly, the odds ratios of developing delirium with high expression levels were 4.32 (*PER2*, 95% CI 1.14–13.87) and 4.5 (*HO1*, 95% CI 1.23–14.42), respectively. Patients classified as delirious according to their maximum nuDesc showed significantly higher SDs of nuDesc during their hospital stay (*P* < 0.0001, Fig. 4g).

**Table 2:** Median *HO1* and *PER2* mRNA expression between groups of TBI/non-TBI and delirium/non-delirium over time.

	n	TBI		Non-TBI			Delirium		Non-delirium		
		24		24			19		29		
		Median	IQR	Median	IQR	*P*	Median	IQR	Median	IQR	*P*
*PER2* expression	Day 1	3.419	[2.38 to 4.085]	2.319	[1.647 to 3.828]	0.125	3.490	[2.830 to 5.927]	2.325	[1.635 to 3.533]	0.013
	Day 8	2.702	[1.808 to 3.829]	2.235	[1.033 to 3.382]	0.322	3.483	[2.388 to 5.477]	1.863	[1.083 to 2.964]	0.016
*HO1* expression	Day 1	5.130	[3.721 to 6.273]	5.634	[3.302 to 10.46]	0.646	6.130	[5.130 to 10.66]	4.371	[2.998 to 6.242]	0.004
	Day 8	3.666	[2.808 to 5.147]	4.053	[3.016 to 5.527]	0.504	4.895	[2.942 to 5.793]	3.583	[2.847 to 4.294]	0.191

Sample acquisition on first day and after 1 week. *P*-values of between-group comparisons of *PER2* and *HO1* mRNA expression (median and IQR) calculated via Mann Whitney test between groups of patients with and without delirium as well as between TBI and non-TBI groups.

*PER2* period circadian regulator 2, *HO1* heme oxygenase 1, *TBI* traumatic brain injury, *IQR* inter-quartile ratio.

Bilirubin concentration neither correlated with maximum nuDesc (r = 0.065, *P* = 0.663, r^2^ = 0.011, *P* = 0.486, data not shown) nor were patients with high bilirubin levels more likely to develop delirium compared to those with low levels (*P* = 0.770, data not shown).

### TBI influences *PER2* expression but not *HO1* expression or occurrence of delirium

**Figure 5:**
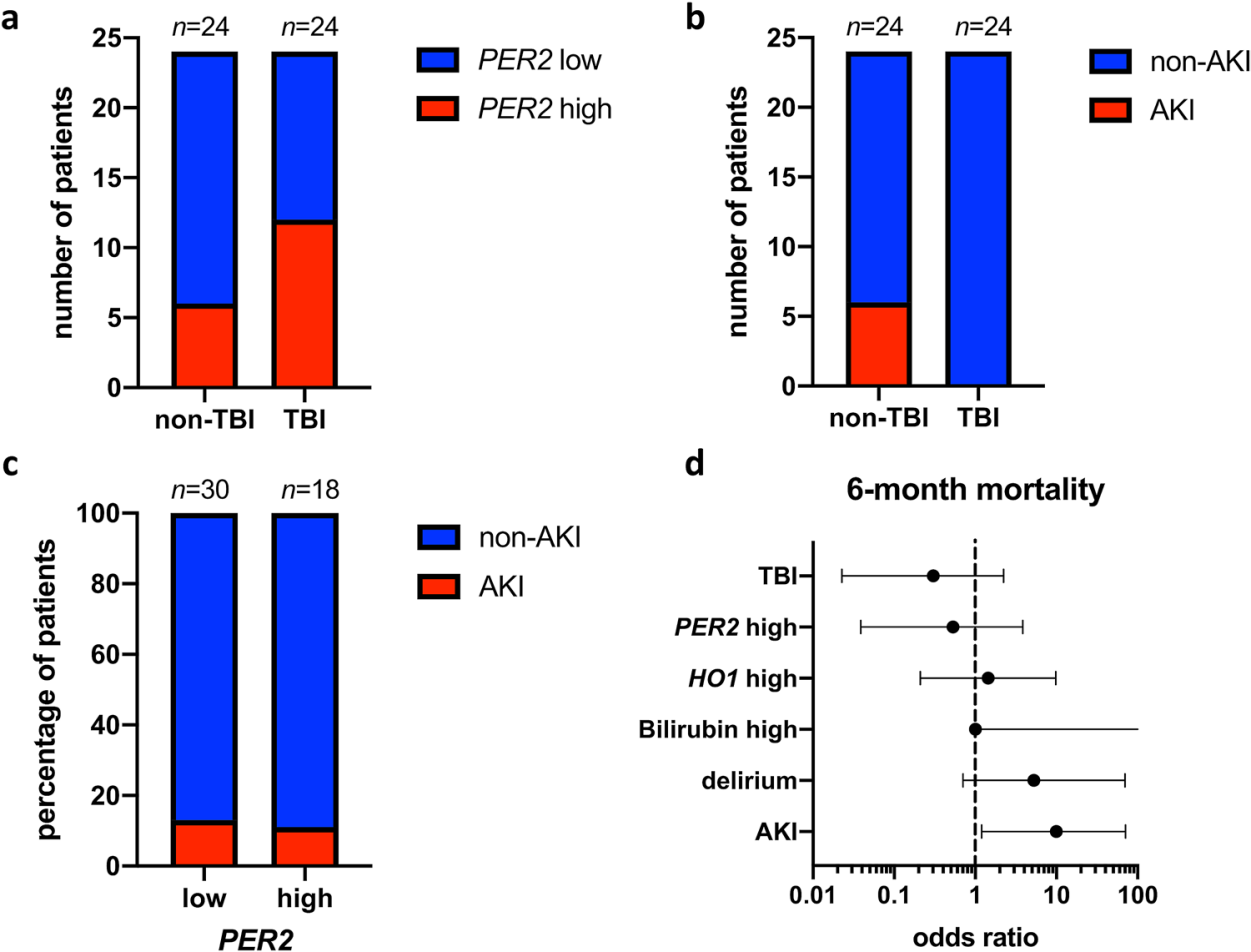
Patient distribution in relation to biomarkers, TBI and mortality. **(a–c)** Contingency diagrams analyzed via Fisher’s exact test showing distribution of **(a)** high/low *PER2* (*P* = 0.135, Koopman asymptotic score: *RR* = 2.00, 95% CI 0.94–4.52; Baptista-Pike: *OR* = 3.00) between patient groups of traumatic brain injury (TBI) and non-TBI as well as **(b)** acute kidney injury(AKI)/non-AKI between patient groups of TBI and non-TBI (*P* = 0.022) and between patient groups of high and low *PER2* (**(c)**, *P* > 0.999). **(d)** Forrest plot of odds ratios (*OR*) of 6-month mortality for the variables of TBI (*OR* = 0.30, 95% CI 0.02–2.23), high *PER2* mRNA expression (*OR* = 0.53, 95% CI 0.04–3.85), high *HO1* mRNA expression (*OR* = 1.44, 95% CI 0.21–9.80), delirium (*OR* = 5.25, 95% CI 0.71–70.17) and AKI (*OR* = 10.00, 95% CI 1.20–70.95). *TBI* traumatic brain injury, *PER2* period circadian regulator 2, *AKI* acute kidney injury, *HO1* heme oxygenase 1, *RR* relative risk, *95% CI* 95% confidence interval, *ISS* injury severity score, *95% CI* 95% confidence interval.

As shown in Table 2, no significant differences in mRNA expressions were apparent for either *PER2* or *HO1* between groups of TBI and non-TBI patients. However, there was a tendency towards elevated values of *PER2* on day one after trauma in TBI patients with a relative risk of 2.00 (95% CI 0.94–4.52) and an *OR* of 3.00 (95% CI 0.84–9.33, *P* = 0.125). A similar non-significant tendency was apparent when comparing the distribution of high and low *PER2* mRNA expression on day one after trauma comparing TBI and non-TBI (*P* = 0.135, Fig. 5a). Distribution of high and low *HO1* mRNA expression and bilirubin concentration showed no difference between TBI and non-TBI patients (*HO1*: *P* = 0.770; bilirubin: *P* = 0.561). Similarly, analysis of delirium occurrence in TBI and non-TBI patients revealed no difference in delirium when comparing TBI with non-TBI groups (*P* = 0.556).

### TBI patients show smaller likelihood of developing AKI

As a second clinical readout, the occurrence of AKI was investigated in relation to the biomarkers defined above and in relation to the presence or absence of TBI. Compared with non-TBI trauma patients, TBI patients showed a significantly lower risk of developing AKI (*P* = 0.022, Fig. 5b). However, higher prevalence of AKI did not significantly coincide with either high *PER2* (*P* > 0.999, Fig. 5c) or *HO1* mRNA expression (*P* = 0.219). Patients with high bilirubin concentrations on day one non-significantly tended towards developing AKI (*P* = 0.073, data not shown).

### Six-month mortality does not differ between TBI versus non-TBI and between biomarker risk groups

Of the 48 trauma patients included in the study, four patients died within 6 months after ICU admission. Descriptive statistical analysis (retrospectively and limited by an underpowered sample size) identified both TBI (*RR* = 0.33, 95% CI 0.049–2.16; *OR* = 0.30, 95% CI 0.023–2.23) and high *PER2* mRNA expression (*RR* = 0.56, 95% CI 0.082–3.53; *OR* = 0.53, 95% CI 0.039–3.85) to be associated with less 6-month mortality, while high *HO1* mRNA expression (*RR* = 1.40, 95% CI 0.26–7.43; *OR* = 1.44, 95% CI 0.21–9.80), high bilirubin concentration (*RR* = 1.31, 95% CI 1.04–1.31; *OR* = Infinity, 95% CI 1.00-Infinity), delirium (*RR* = 4.58, 95% CI: 0.70–30.69; *OR* = 5.25, 95% CI 0.71–70.17) and AKI (*RR* = 7.00, 95% CI 1.32–33.01; *OR* = 10.00, 95% CI 1.20–71.00) carried an elevated risk of death (Fig. 5d). However, each of these associations remained non-significant (TBI: *P* = 0.609, high *PER2*: *P* > 0.999, high *HO1*: *P* > 0.999, high bilirubin: *P* = 0.103, delirium: *P* = 0.286, AKI: *P* = 0.071).

## Discussion

In this study, we measured systemic *PER2* and *HO1* mRNA expression on day one after trauma. We conclude that both *HO1* and *PER2* may serve as novel blood biomarkers for trauma severity, length of ICU-stay and, most interestingly, the risk of developing trauma-associated delirium.

There is an unmet need for objective laboratory parameters to improve diagnosis and treatment algorithms for delirium. Many have been tested but have so far proven incapable of having diagnostic value in a clinical setting^11^. Delirious patients commonly demonstrate compromised sleep–wake-cycles, and molecular circadian disruption is considered to play a major role in delirium pathogenesis^2^. Nonetheless, circadian gene expression has not been investigated as potential biomarkers for delirium. Our data suggests that *PER2* and *HO1* mRNA expressions may prospectively assess delirium and trauma severity. Further we measured concentrations of bilirubin, the final product of heme degradation by HO1. By demonstrating a correlation between *HO1* expression and bilirubin concentration we can confirm the notion that mRNA expression mirrors actual enzymatic activity.

Expression of *PER2* was not only significantly elevated on day one, but remained elevated one week post trauma. These findings suggest not only predictive value, as is the case with *HO1*, but also provided diagnostic value for occurrence and severity of trauma-associated delirium. Although the patient’s age at trauma is a known major risk factor for developing delirium^29^ it could not explain the predictive aspects of *PER2* and *HO1*, suggesting age-independence and hence potentially additive diagnostic value. Trauma severity did not contribute to identifying patients at risk for trauma-associated delirium.

Although circadian disruption is thought to contribute to kidney disease^3,4^, no predictive or diagnostic capacity of *PER2* or *HO1* expression could be found for AKI. However, this may be due to the limited amount of AKI cases in our patient cohort. Work by others strongly suggests that *HO1* plays a major role in stress response to acute kidney injury^21–26,30^. Indeed, patients with more GT-repeats in the *HO1*-gene promoter region have been shown to be less likely to develop AKI^30^. Therefore, further studies should investigate the predictive and diagnostic capacity of *HO1* and *PER2* for those with AKI.

Similarly, 6-month mortality could not be attributed to high *HO1* and *PER2* expression as well as TBI. With a total of only four deceased patients, our cohort of n = 48 was underpowered for this endpoint and similar to AKI, larger cohort studies might prove to yield more conclusive results. On a descriptive statistical level limited by our patient cohort, our data suggest that TBI and high *PER2* expression may be favorable for survival, while high *HO1* expression, high bilirubin, delirium and AKI carried a greater risk of death. The last two of which are already considered to contribute to increased mortality^5,6^.

Intracranial bleeding has been shown to cause disruption in circadian gene expression in mice^20^. It was investigated whether TBI has an effect on the likelihood of developing delirium and AKI. TBI did not show any significant impact on occurrence of trauma-associated delirium or systemic mRNA expression levels of *PER2* and *HO1*. Thus, our findings cannot support our hypothesis of TBI on top of polytrauma causing more severe molecular circadian disruption and thereby contributing to the pathogenesis of delirium and AKI. It is possible, however, that our definition of TBI as a radiographical diagnosis might have been too broad. Our categorization may not have allowed for sufficient effect sizes and significant differences between TBI and non-TBI groups. In addition, systemic changes in mRNA expression were measured in peripheral blood leucocytes, which might not be representative of the regional changes in *PER2* and *HO1* expression in brain tissue following TBI. TBI patients tended to have elevated *PER2* expression on day one after trauma when compared with non-TBI patients. Further studies evaluating regional *PER2* and *HO1* expression following TBI, e.g. in the cerebrospinal fluid might help fill this gap.

Interestingly, PER2 expression in peripheral blood cells as a surrogate for disturbance of circadian rhythmicity has recently been studied in both polytrauma and general ICU patient collectives^31,32^. In both cases, low PER2 expression was associated with systemic inflammation and thereby may mirror progression towards multi-organ dysfunction syndrome^33^. Strikingly, in our patient cohort the likelihood of AKI was significantly reduced in TBI patients compared with the non-TBI control group while non-significantly tending towards elevated *PER2* expression. Taking into account work by others concerning nephro-protective qualities of *PER2*^34^, along with our findings, it is tempting to speculate that TBI induces *PER2* expression, which on the one hand indicates central circadian disruption that contributes to the development of delirium while on the other hand exerting anti-inflammatory organ protection peripherally by preserving kidney function. Fittingly, on a descriptive statistical level and limited to our patient cohort, TBI and *PER2* induction showed a trend towards a beneficial impact on survival.

Several limitations of this study have to be noted in relation to the findings discussed above. First, our patient sample size was limited and restricts generalizability of our findings. Second, the ICU as a “polluted” environment in terms of external circadian entrainment factors poses challenges for making conclusions about true endogenous circadian signaling. Third, we performed static analysis of circadian gene expression at two time points that might not reflect true circadian oscillation of these genes.

## Conclusions

In summary, systemic mRNA expression of *PER2* and *HO1* is induced after trauma that correlates with trauma severity. Furthermore, the extent of expression correlates with the incidence of delirium in an age-independent manner. TBI in our cohort did not contribute to delirium development. However, it was associated with reduced incidence of AKI, suggesting a link between TBI and secondary organ injury. Utilizing expression of *PER2* and *HO1* as biomarkers could potentially improve diagnosis algorithms for delirium by adding an objective laboratory parameter.

## Methods

### Study

This observational biomarker study focused on TBI in the setting of polytrauma and regulation of *PER2 and HO1* expression in leucocytes along with ICU-relevant clinical outcome data (Fig. 1a). In this single-center cohort study, patients were studied under a protocol that was approved by the Institutional Ethics Committee of the University of Freiburg (Protocol No. 293/15). All procedures performed in studies involving human participants were in accordance with the ethical standards of the institutional and/or national research committee and with the 1964 Helsinki Declaration. Data reporting is done in accordance with the STROBE guidelines. Informed consent from the patient, legal guardian or by proxy was provided. The trial was registered with the German Clinical Trials Register (Trial-ID DRKS00008981; Universal Trial Number U1111-1172-6077; first registration Jul 31, 2015; amendment Jan. 18, 2018).

### Patients and procedures

Between 13.07.2018 and 06.08.2019, patients who were admitted to the ICU in the Department of Anesthesiology & Critical Care Medicine, University of Freiburg, were screened.

Inclusion criteria:

1. Adult patients with polytrauma (ISS > 16) ± TBI.
2. Admission to ICU within 24 h after trauma.
3. Blood sample acquisition possible within 24 h after admission.
4. Possibility to receive informed consent from the patient, legal guardian or by proxy.

Exclusion criteria:

1. Pre-existing kidney disease or pre-existing impaired kidney function.
2. Pre-existing diagnosed dementia.
3. Admission later than 24 h after trauma.
4. Death of patient within 24 h after admission.
5. Traumatic or non-traumatic brain injury within 14 days prior to admission.

During the recruitment process, two patient groups (TBI versus non-TBI) were created, limiting differences in gender, age and trauma severity (ISS) using a matched pairs approach. For this purpose, three sub-groups for both age and trauma severity were defined: (1) patients younger than 40 years, (2) patients between 40 and 75 years and (3) patients older than 75 years. These were further grouped by trauma severity into those with an ISS of 24, an ISS of 25 and 36 and an ISS of > 36. Patient groups did not differ in any of the sub-group categorizations (cf. Table 1). TBI was defined as a radiographically diagnosed brain trauma in a routine trauma cranial computer tomogram performed upon hospital admission. Data for clinical assessment was obtained from the digital patient file. Patient mortality was determined from either the digital patient file or via contact with the patients and/or their relatives six months after admission.

### Clinical assessment

Delirium was assessed by trained ICU staff every 8 h using the nursing delirium screening scale (nuDesc)^35^. In patients with deep sedation for therapeutic reasons, delirium screening was resumed as soon as sedation was terminated. Maximum nuDesc scores during the hospital stay were used to correlate with biomarker expression. To perform between-group comparisons patients were classified into groups of “delirium” and “non-delirium” according to their maximum nuDesc. However, the maximum nuDesc is a static evaluation of delirious symptoms and may not be specific to delirium alone when applied at a single point of time. Therefore, the classification was validated based on a dynamic criterion of delirium that is mentioned in the DSM-5 (fifth edition of the Diagnostic and Statistical Manual of Mental Disorders): fluctuation of delirious symptoms. Symptom fluctuation was assessed through SD (standard deviation) of all daily nuDesc values during patient’s hospital stay. This SD was then defined as the independent variable in a between-group comparison of “delirium” and “non-delirium” via Mann–Whitney-U test. Acute kidney injury was assessed using the first KDIGO (kidney disease improving global outcomes) criterion (0.3 mg/dl serum creatinine increase within 48 h (Stage 1)). The injury severity score (ISS) was obtained from the emergency ward admission sheet that is routinely assessed in polytrauma patients by the admitting staff.

### Biomarkers

Blood was drawn from arterial or venous catheters during the morning hours on the first day (within 24 h after admission) and one week after trauma (Tempus Blood RNA Tube, AB#4342792). RNA isolation from leucocytes via spin-column purification (Tempus Spin RNA Isolation Kit, AB#1710145) was done as recommended by the manufacturer. Equal amounts of RNA were reversely transcribed into cDNA via reverse transcriptase PCR (iScript cDNA Synthesis Kit, BioRad#1708890; PeqStar 96 Universal Gradient, PeqLab), followed by semi-quantification via real-time PCR (StepOnePlus Real Time PCR-System, A&V Applied Biosystems) with nucleic acid stain (PowerUp SYBR Green Master Mix, AB#1708020).

Primer Sequences were:

**Table:** 

*PER2* forward	TCCTCGGCTTGAAACGGC
*PER2* reverse	GAACGAAGCTTTCGGACCTCA
*HO1* forward	GTGATAGAAGAGGCCAAGACTG
*HO1* reverse	GAATCTTGCACTTTGTTGCTGG
*Rpl13a* forward	CGGACCGTGCGAGGTAT
*Rpl13a* reverse	CACCATCCGCTTTTTCTTGTC

Gene expression analysis was performed relatively using ΔΔCt-method. Rpl13a served as reference gene as it provides stable expression profiles in various tissues and cell types including peripheral blood and across various experimental conditions^36–39^. Lacking pre-trauma sampling, cDNA samples of five healthy individuals without a history of trauma or known preconditions were recruited in order to obtain normal baseline expression patterns. These pooled technical baseline values were than used to calculated relative changes in mRNA expression in trauma patients.

Furthermore, maximum plasma concentrations of bilirubin (as the final product of heme degradation) from blood gas analyses were obtained from the digital patient file matching the corresponding time of blood withdrawal for gene expression analysis. Plasma bilirubin quantification was done by direct spectrophotometry using a blood gas analyzer (ABL 800 Flex, Radiometer).

### Statistics

Data analysis was done using Prism 8 software (GraphPad Software Inc.). Based on previous data^40^, an a priori power analysis (contingency tables *PER2/HO1* high/low vs. delirium/non-delirium; effect size Cohen’s w 0.6; α = 0.05; power 95%, df 2) indicated a total sample size of *n* = 43 to be sufficient to characterize biomarker expression levels. Patients for whom data on outcomes were missing were excluded from the study. When performing measurements of central tendency in ordinal-scaled data (*PER2* expression, *HO1* expression, bilirubin concentration, maximum leucocyte count of first week) the median value is presented along with the corresponding IQR (inter-quartile ratio). In instances of cardinal-scaled data (age, ISS (injury severity score), time on ICU) the mean value is presented with SD. Between-group differences in metrical data (age, ISS, time on ICU, *PER2* expression, *HO1* expression, bilirubin concentration, maximum leucocyte count of first week) were analyzed via Mann–Whitney-U test. Between-group comparisons in categorical data were performed using Fisher’s exact test (yes/no: pre-existing neurological disease, pre-existing cardiovascular disease, diabetes mellitus, TBI, delirium, AKI, 6-month mortality; high/low: *PER2* expression, *HO1* expression, bilirubin concentration). Tests for correlation were performed between metrical data sets (ISS, *PER2* expression, *HO1* expression, bilirubin concentration, time on ICU, age, maximum nuDesc) using Spearman correlation (r, *P*) as well as linear regression (r^2^, *P*). Categories of high and low expression levels of *PER2* and *HO1* were formed from linear regression of ISS and mRNA expression levels of *PER2* or *HO1* as well as maximum bilirubin concentration on day one after trauma. A linear regression formula of the format Y = a × x + b with the corresponding curve was calculated. Then, data points with mRNA expression/bilirubin concentration located above the regression curve, mathematically corresponding to values higher than expected when using the regression formula for the individual data point, were defined to be of high expression/concentration. Data points with mRNA expression/bilirubin concentration located below the regression curve, mathematically corresponding to values lower than expected using the regression formula, were defined to be of low expression/concentration. Descriptive statistics of relative risk (*RR*) and odds ratio (*OR*) were assessed via Koopman asymptotic score (*RR*) and Baptista-Pike (*OR*). *RR* and *OR* are presented along with their 95% confidence interval (95% CI). With all statistical analyses, a *P*-value of less than 0.05 was considered to be significant. A *P*-value of less than 0.15 was considered to be indicative of a possible association, and is referred to as “non-significant tendency”.

## Data Availability

The data that support the findings of this study are available from the corresponding author upon reasonable request.
